# Macroclimatic Convergence and Habitat Specialisation Shape the Mediterranean Seed Germination Syndrome

**DOI:** 10.1002/ece3.70527

**Published:** 2024-11-07

**Authors:** Diana María Cruz‐Tejada, Efisio Mattana, Sergey Rosbakh, Eduardo Fernández‐Pascual, Angelino Carta

**Affiliations:** ^1^ Botany Unit, Department of Biology University of Pisa Pisa Italy; ^2^ Royal Botanic Gardens, Kew Ardingly UK; ^3^ Department of Plant and Environmental Sciences University of Copenhagen Denmark; ^4^ Biodiversity Research Institute (IMIB) University of Oviedo, CSIC, Principality of Asturias Mieres Spain; ^5^ CIRSEC, Centre for Climate Change Impact University of Pisa Pisa Italy

**Keywords:** habitat specialists, macroclimate, macroecology, macroevolution, Mediterranean syndrome, seed germination

## Abstract

Ecological theory predicts that plant reproductive phenology in the Mediterranean regions is shaped by evolutionary processes driven by strong seasonality in precipitation–evaporation patterns. Thus, it can be expected that seed germination phenology has adapted to maximise recruitment during the season of highest water availability. Cold‐cued and slow germination (i.e., the ‘Mediterranean seed germination syndrome’) has been hypothesised to be an adaptation to ensure that seedling emergence occurs in autumn/early winter, extending the growing season before the subsequent unfavourable summer drought. However, this hypothesis has been tested on individual species or local studies, without a proper synthesis for the whole Mediterranean region. Here we tested, for the first time, the Mediterranean seed germination syndrome using experimental data for 459 species (11,363 records, 59 families) occurring in the Mediterranean Basin. We performed a phylogenetically informed Bayesian meta‐analysis to model the effect on germination proportions of seven key experimental cues: mean incubation temperature, alternating temperature regime, light and dormancy‐breaking treatments (scarification, warm stratification and cold stratification) and the modulating role of seed mass on seed germination. We show that species from lowland zonal habitats of the Mediterranean align with the Mediterranean germination syndrome hypothesis, with their seeds responding positively to cool, constant temperatures and negatively to light. Yet, habitat specialists (i.e., species restricted to mountains, coasts and wetlands) deviate from the syndrome, showing nearly opposite germination requirements. Seed mass further influences the germination niche and phylogenetically related species exhibit similar germination responses. Cumulatively, these results suggest that evolutionary pressures from local habitat‐related conditions override the macroclimatically imposed Mediterranean seed germination syndrome.

## Graphical abstract

Ecological theory predicts that plant reproductive phenology in the Mediterranean regions is shaped by evolutionary processes driven by strong seasonality in precipitation–evaporation patterns. Here we tested, for the first time, the Mediterranean seed germination syndrome using experimental data occurring in the Mediterranean Basin. We show that species from lowland zonal habitats of the Mediterranean align with the Mediterranean germination syndrome hypothesis while habitat specialists deviate from the syndrome, showing nearly opposite germination requirements. Cumulatively, these results suggest that evolutionary pressures from local habitat‐related conditions override the macroclimatically imposed Mediterranean seed germination syndrome.

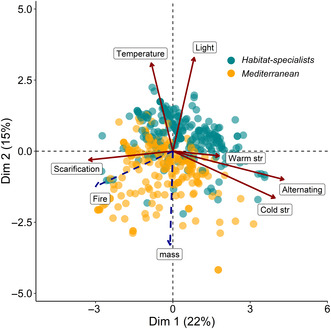

## 1 Introduction

To ensure successful regeneration, dispersed propagules must germinate at the right place and at the right time (Baskin and Baskin 2014; Pausas et al. 2022). As such, seed germination is a critical stage in the life cycle of plants that occurs as a response to a combination of environmental cues (Bewley et al. 2013; Baskin and Baskin 2014) constituting the ‘seed germination niche’ (Grubb 1977; Donohue et al. 2010; Carta et al. 2022). This niche captures the combination of environmental cues (including dormancy‐breaking conditions and germination requirements) under which a species can complete a successful transition from seed to seedling, ensuring seedling survival and growth (Grubb 1977). Previous research has demonstrated that the germination niche is shaped by macroclimate and local habitat conditions (Fenner and Thompson 2005; Baskin and Baskin 2014; Fernández‐Pascual et al. 2021; Carta et al. 2022). Nonetheless, the germination niche varies nonrandomly across the phylogeny, with closely related species often exhibiting similar niches (Carta et al. 2022) and is also influenced by the seed mass (Zhang et al. 2020; Carta et al. 2022), which is a key trait in the regeneration stages (Thompson and Grime 1983; Pons 2000). Correlations between seed germination requirements and climatic conditions suggest that the germination niche is shaped by evolutionary pressures at macroclimatic scales with germination phenology adapted to maximise recruitment during the appropriate time and place (Donohue et al. 2010; Baskin and Baskin 2014; Carta et al. 2022). This is of pivotal importance in markedly seasonal biomes, like the Mediterranean climatic regions of the world (Mattana et al. 2022).

The Mediterranean regions, covering only 2% of Earth's land surface, host approximately one‐sixth of the world's vascular plant flora (Rundel et al. 2018) and are considered a global biodiversity hotspot (Myers et al. 2000). The Mediterranean biome is present in different geographic regions (California, South Africa, central Chile, southern Australia and the Mediterranean Basin) that share a common macroclimate determined by mild wet winters and warm dry summers (Rundel et al. 2018), frequent natural wildfires (Keeley and Pausas 2019) and lowland vegetation characterised by sclerophyllous plants (Joffre, Rambal, and Damesin 2007). Among these regions, the Mediterranean Basin is also characterised by a complex geography, resulting in considerable habitat diversity (Lionello et al. 2006). This habitat diversity is further influenced by historical human activity going back thousands of years (Thompson 2020), which in turn, has shaped Mediterranean ecosystem dynamics (Médail and Quezel 1999; Hernández Fernández, Álvarez Sierra, and Peláez‐Campomanes 2007; Barton, Ullah, and Bergin 2010).

The strong and seasonal water limitation that drives evolutionary processes in the Mediterranean has been suggested to shape seed germination phenology to maximise recruitment during the season of highest water availability. Several adaptations (e.g., slow germination under cold temperatures), matching germination with the winter wet season (Thanos, Kadis, and Skarou 1995; Doussi and Thanos 2002), have been long conceptualised in the literature as the Mediterranean seed germination syndrome. Other features of the Mediterranean seed germination syndrome include the photoinhibition of seed germination (Thanos et al. 1991), a mechanism that detects depth and suppresses germination near the dry soil surface (Carta et al. 2017) and the lack of a diurnal fluctuation temperature detection mechanism (Saatkamp et al. 2011). Moreover, seeds of many species from the Mediterranean show adaptations to wildfires (Keeley 1986; Moreira et al. 2010), which are a natural environmental factor that can act as a signal to terminate physical dormancy (i.e., impermeable seed coats; Thanos and Georghioi 1988; Pausas and Lamont 2022; Luna et al. 2023). Fire‐related germination is especially common in Cistaceae and Fabaceae species, which show a positive germination response to heat shock and smoke (González‐Rabanal and Casal 1995; Moreira et al. 2010; Kazancı and Tavşanoğlu 2019). Nonetheless, positive responses are found also in plants from other families, not strictly related to postfire regeneration (Buhk and Hensen 2006; Luna et al. 2007). Another aspect of the Mediterranean germination syndrome is that species experience warm dry conditions after dispersal, leading to physiological dormancy release by dry after‐ripening in summer which synchronises germination with autumn rainfall (Baskin and Baskin 2014).

However, these functional adaptations may not benefit all Mediterranean plants, as some species might be locally exposed to different selective pressures. For instance, mountain Mediterranean plants suffer less water limitation and are exposed to colder winters than species from the lowlands. Meanwhile, other local habitat specialists, such as coastal and wetland species, live in areas where the environmental conditions further deviate from the Mediterranean macroclimatic pattern. Consequently, it can be expected that the seed germination responses of these species do not comply with the Mediterranean seed germination syndrome hypothesis. Particularly, some studies have reported that cold stratification, light conditions and higher temperatures could be beneficial for the germination of local habitat specialists living in the Mediterranean, including mountain, coastal and wetland species (Giménez‐Benavides, Escudero, and Pérez‐García 2005; Mattana et al. 2012; Carta et al. 2014; Picciau et al. 2019; Del Vecchio et al. 2020). However, all these studies focused on a limited number of species and a narrow geographical focus.

Despite recent attempts to synthesise seed germination knowledge across major temperate biomes (Fernández‐Pascual et al. 2021; Zhang et al. 2021; Carta et al. 2022), current assumptions about the Mediterranean seed germination syndrome are mainly based on local studies, with limited geographical and phylogenetic scales preventing a proper synthesis for the whole Mediterranean region. To close this gap, we tested the Mediterranean seed germination syndrome through a phylogenetically informed Bayesian meta‐analysis of primary germination proportion data (Carta et al. 2022), by focusing on widely distributed characteristic species of Mediterranean habitats (Chytrý et al. 2020; Cruz‐Tejada et al. 2024). Furthermore, we compared the germination responses of species from lowland zonal habitats and habitat specialists (i.e., species restricted to mountains, coasts and wetlands), expecting that the former group will exhibit the Mediterranean syndrome, while the latter will deviate from it. Specifically, we tested the following predictions derived from the Mediterranean germination syndrome hypothesis: seeds (1) require low temperatures to germinate; (2) show photoinhibition of germination; (3) do not require alternating temperatures for germination; (4) respond negatively to cold stratification; (5) respond positively to warm stratification; (6) need scarification to alleviate dormancy; and (7) respond positively to fire stimuli.

## 2 Material and Methods

### 2.1 Data Collection

#### 2.1.1 Data Set Construction

We compiled a checklist of characteristic Mediterranean species using the European habitat classification system, EUNIS (Chytrý et al. 2020; Cruz‐Tejada et al. 2024). As such, the list comprises species representative of the whole Mediterranean region, its habitat variability and associated ecological processes. We considered the Level 3 EUNIS habitats (i.e., in detail classified European habitats based on species composition, macroclimatic association and geographic location), to distinguish: (1) ‘*Mediterranean’* species as those that are characteristic of lowland zonal habitats where the Mediterranean macroclimate is the major driver of vegetation processes, from (2) ‘*habitat specialists*’ occurring in other Mediterranean habitats such as mountains, coastal habitats and wetlands (i.e., vegetation type azonality, Chytrý et al. 2020) (Table S1). We then standardised all species names against The World Checklist of Vascular Plants, WCVP v10 (Govaerts 2022) using the u.taxonstand package (Zhang and Qian 2022) in R statistical environment (R Core Team 2022). For the species in our checklist, we extracted primary germination data and laboratory experimental conditions provided by SeedArc (Fernández‐Pascual et al. 2023), which includes data extracted from the literature (e.g., MedGermDB: Cruz‐Tejada et al. 2024, SylvanSeeds: Fernández‐Pascual 2021), from seed bank databases (e.g., ENSCOBASE: http://enscobase.maich.gr/), the Alpine data set (Fernández‐Pascual et al. 2021) and personal data (Rosbakh, unpublished data). We included all species with at least one germination record from a seed lot collected in the Mediterranean Basin (e.g., to capture species variability), removing all the species whose seed collection was only outside the Mediterranean (e.g., avoiding external environmental factors that could influence the analyses).

The final data set used in the analyses contained 11,363 records (i.e., germination proportions for a given seed lot of a species, recorded in a set of experimental conditions) from 2888 seed lots from 59 families and 459 species retrieved from 271 sources, representing major Angiosperm clades (Figure S1). From these, 218 species (5267 records) were classified as lowland *Mediterranean*, and 241 species (6096 records) were classified as *habitat specialists* (i.e., 53 species from Mediterranean mountains habitats, 70 species from Mediterranean coastal habitats, 18 species from Mediterranean wetlands and 100 species from other habitats). Each record corresponded to one germination test and included information on the number of sown and germinated seeds, plus the associated seed lot metadata: species standardised name, literature source (i.e., institution or seed bank where the test was carried out) and collection country.

#### 2.1.2 Experimental Germination Cues

To test our hypotheses, we considered laboratory experimental cues simulating environmental conditions (Figure S2), at which each germination test extracted from SeedArc was conducted [mean incubation temperature, temperature regime (alternating vs. constant), light conditions (diurnal light vs. darkness), dormancy‐breaking treatments (cold and warm stratification, scarification) and experimental fire conditions (exposure to heat over 50°C and/or less than 1 h)].

#### 2.1.3 Phylogenetic Tree

We compiled the phylogenetic tree for all species included in the data set using the R package U.PhyloMaker (Jin and Qian 2022). This package contains a megatree based on the updated GBOTB phylogeny for seed plants (Smith and Brown 2018), matching the most taxa of the data set and attached the species absent in the megatree (88 species) to their genus node.

#### 2.1.4 Seed Mass of the Study Species

We gathered the mean seed mass for each species from Carta et al. (2022) to use as a covariate in the models. For some species that were missing seed mass (*n* = 36), we calculated genus averages (as in Fernández‐Pascual et al. 2021).

### 2.2 Statistical Analyses

#### 2.2.1 Meta‐Analysis

We performed a meta‐analysis by fitting binomial phylogenetic generalised mixed models with Bayesian estimation (MCMCglmms) implemented in the R package MCMCglmm (Hadfield 2010) to examine the effects of germination conditions (i.e., environmental cues as fixed effects) on final germination proportion (Carta et al. 2022). Random effects considered were: (1) the phylogenetic tree for the 459 species to account for evolutionary relationships; (2) species identity to address intra and interspecific variation; (3) seed lot and (4) study (i.e., data source) to address seed lot variation and provenience of data, respectively; (5) experiment id; and (6) germination substrate (e.g., agar and filter paper) to address variation in different experiments for the same species.

We tested the interactive effect of the germination treatment and the species type explained above (*Mediterranean* or *habitat specialists*). We also included seed mass as an interactive term to assess its potential impact on seed germination. For detailed numerical results for all models, see Table S2. Model summaries include information on the fixed and random effects (Table S2). Fixed effects represent how the predictive variables (i.e., experimental cues) affect the response variable (i.e., final germination proportion). Specifically, we tested the main effect of experimental cues on seed germination and the interactive effect of the experimental cues and species type (i.e., *Mediterranean* vs. *habitat specialists*).

All analyses were run across the full data set including all the species, and separately, for the largest clades: monocots, rosids, asterids (Table S3 and Figure S3). While all models accounted for the species phylogenetic relatedness (as a random factor), running the same models separately for each clade allowed to visualise whether the seed germination responses were the same across clades, or if there were lineage‐specific responses. All models were run with weakly informative priors, with parameter expanded priors for the random effects. Each model was run for 500,000 MCMC steps, with an initial burn‐in phase of 50,000 and a thinning interval of 50. Model parameters were determined based on mean estimates and 95% credible intervals (CIs). Parameters with CIs overlapping zero were considered nonsignificant.

#### 2.2.2 Visualisation of the Mediterranean Seed Germination Syndrome

To visualise the seed germination niches, we applied a multiple factor analysis (MFA) using the FACTOMINER package (Le, Josse, and Husson 2009). This ordination method combines principal component analysis (PCA) and multiple correspondence analysis (MCA) to capture variation in both continuous (germination traits and seed mass) and categorical variables (*Mediterranean* vs. *habitat‐specialist* species groups). Since the available data for experimental fire conditions were not well represented in the data set, we included this cue as a supplementary variable not contributing to the construction of the ordination. We also included seed mass as a supplementary variable as we were interested to understand its covariation with the germination niche (see Le, Josse, and Husson 2009 and Carta et al. 2022 for details on using supplementary variables). For the ordination, germination traits were aggregated at the species level, and the final germination proportion was transformed by averaging cue levels (e.g., temperature) with binary variables (absence = 0, presence = 1) to create a continuous variable for each germination cue (see Fernández‐Pascual et al. 2021 and Carta et al. 2022 for details on this procedure). It is essential to note that this approach served only for data visualisation and not for statistical inference (which is provided by the models).

## 3 Results

### 3.1 Meta‐Analysis Models

**FIGURE 1:**
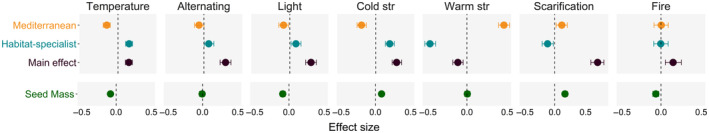
Effect of the experimental cues on final germination proportions according to the MCMC meta‐analysis of primary data. Dots indicate the posterior mean of the effect size, and whiskers its 95% credible interval. The line of zero effect is shown. When the credible intervals overlap with the zero‐effect line, the effect is not significant. Some effects that overlapped with zero (i.e., had no effect) and whose credible intervals were excessively wide are not shown for clarity's sake. The figure shows the effect of experimental cue on seed germination in *Mediterranean* lowland species (orange), in *habitat specialists* (Turkish blue) and the main effect (dark). The dark green point indicates the interactive effect of seed mass. A negative effect indicates that the response to the experimental cue (e.g., temperature) decreases when the mean temperature (environmental cue) is high.

Our models revealed that higher germination temperatures, alternating temperature, light and cold stratification had negative effects on the seed germination of *Mediterranean* species, as shown by credible intervals not overlapping zero (Figure 1). Warm stratification and scarification had a positive effect on seed germination, while fire‐cues had no significant effects. These results indicate that, overall, the germination of Mediterranean plants is promoted by low and constant temperatures, absence of light, warm stratification and scarification. However, the germination niche of *habitat specialists* deviates from the general Mediterranean syndrome, showing opposite seed germination responses for most of the experimental cues (Figure 1). When analysing all the species together (main effects in Figure 1) the pattern for *habitat specialists* dominates and germination is positively and significantly associated with most experimental cues (i.e., temperature, alternating, light, cold stratification, scarification and fire).

The values of seed mass ranged from 0.008 mg (*Neottia nidus‐avis* (L.) Rich.) to 4000.51 mg (*Quercus cerris* L.), with a median value of 32.33 mg, *Mediterranean* species had higher mean seed mass compared with *habitat specialists* species (*t* = 21.484; *p* < 0.01). Seed mass has a significant negative interaction with germination temperature, light and fire and a positive interaction with scarification and cold stratification (Figure 1). Thus, the germination of heavier seeds was reduced at higher temperatures in the light, but was positively affected by experimental fire conditions, scarification and cold stratification.

These patterns are consistent across all main Angiosperm clades (Figure S3) with few exceptions (e.g., asterids) indicating homogeneity in the seed germination responses of Mediterranean plants to different experimental conditions. For instance, warm stratification mainly promoted the germination of rosids and asterids species. Rosids and asterids showed marked preferences for constant temperatures, while monocots showed opposite trends, and rosids showed significant and positive effects to fire.

### 3.2 Multiple Factor Analysis

**FIGURE 2:**
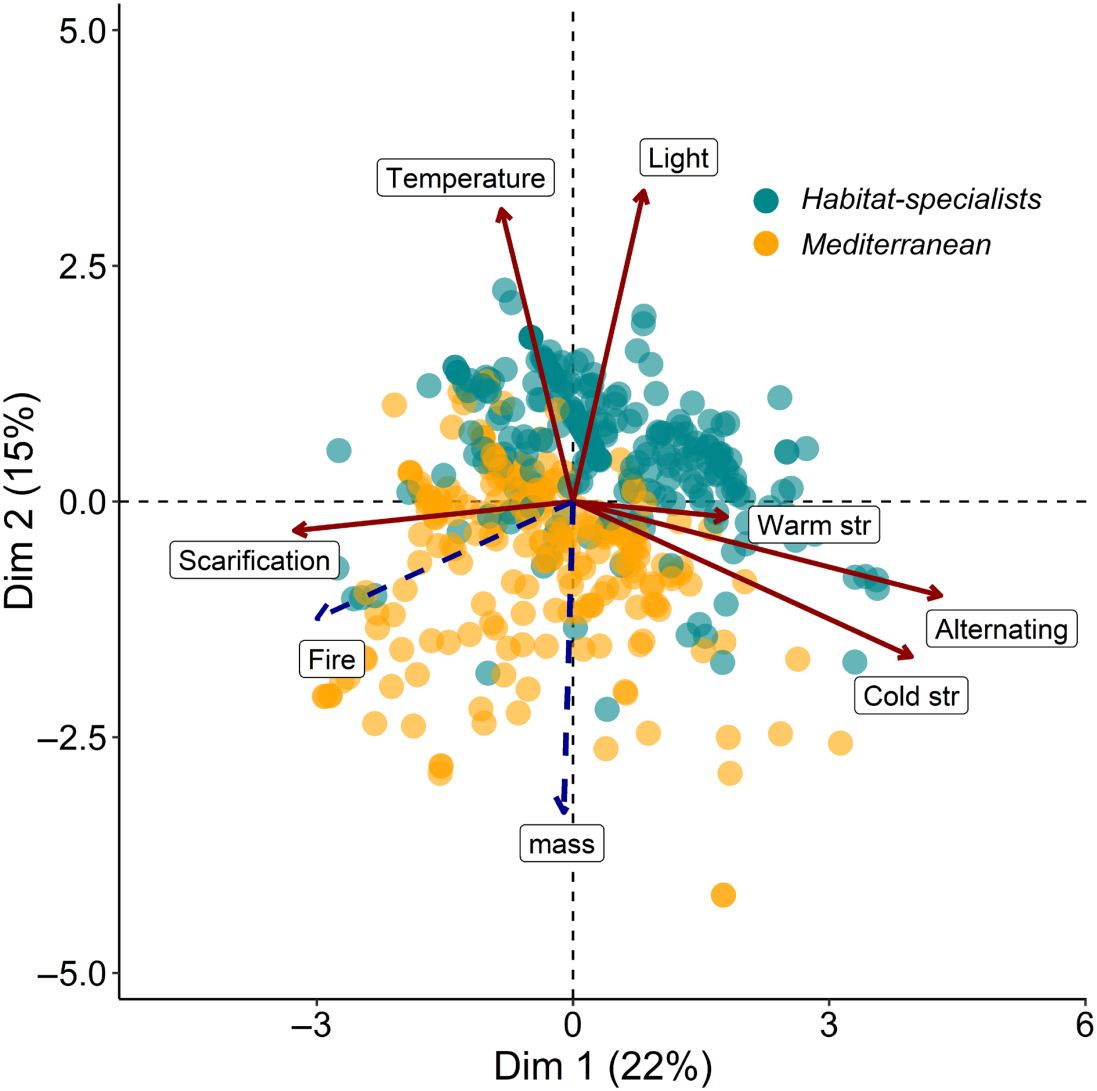
Visualisation of the seed germination niche of the Mediterranean plants through a Multiple Factor Analysis (MFA) summarising the cross‐covariance between seed germination traits and seed mass along the first two axes. Each dot is a species coloured by its habitat type (as indicated). Solid arrows correspond to loadings of the seed germination experimental conditions used to construct the ordination. Dashed arrows correspond to supplementary variables, not used to construct the ordination.

The first MFA axis (22% variation explained; Figure 2) aligned with germination responses to alternating temperatures, cold stratification, warm stratification and scarification. This axis separated species showing positive responses to scarification (left) from species with responses to alternating temperatures, cold stratification and warm stratification (right). The second MFA axis (15% variation explained) aligned with germination responses to light and temperature and separated *Mediterranean* species with cold‐cued germination in absence of light (bottom) from *habitat specialists* with warm‐cued germination and a requirement for light (top). Fire covaried with the first axis, while seed mass covaried with the second one, indicating that *Mediterranean* species have heavier seeds than the *habitat specialists*. In general, the MFA shows that while the horizontal axis indicates common responses across all species, the vertical axis reflects distinct germination responses of *Mediterranean* species which exhibit the Mediterranean seed germination syndrome, showing an overall agreement with the meta‐analytical models reported above. The MFA did not allow to identify specific combination of germination responses between clades (Figure S4), suggesting that, in general, species across the phylogeny share seed germination responses regardless of the clade they belong to.

## 4 Discussion

### 4.1 Definition of the Mediterranean Seed Germination Syndrome

Our study is the first formal test of the Mediterranean germination syndrome. We demonstrate that this syndrome is characterised by cool temperatures, photoinhibition of seed germination, wildfire adaptations, positive responses to scarification and warm stratification, and an interplay with seed mass (e.g., larger seeds align their germination with the Mediterranean syndrome). These germination responses are consistent across phylogenetically distant species, suggesting that the seed germination of Mediterranean species is subjected to convergent evolution mediated by pressures at the macroclimatic level. However, our analysis showed that this syndrome is exhibited only by *Mediterranean* species from lowland zonal habitats, while local *habitat specialists* exhibited opposite germination requirements.

The Mediterranean seed germination syndrome is exhibited by species characteristic of *Mediterranean* lowland zonal habitats (e.g., annual grasslands, shrublands and evergreen oak forests), where the Mediterranean seasonal drought is the major evolutionary pressure (Thompson 2020). The germination of these species is stimulated by cool temperatures (Thanos, Kadis, and Skarou 1995; Doussi and Thanos 2002), favouring plant regeneration by seeds during the cool seasons, when water stress is the lowest, ensuring seedling emergence and prolonging the growing season before the harsh summer drought (Thanos, Kadis, and Skarou 1995). Moreover, germination suppression by light is a mechanism that helps to prevent germination near the soil surface, where conditions in the Mediterranean can be dry and unfavourable for seedling establishment (Carta et al. 2017). At the same time, this mechanism works in conjunction with the diurnally varying temperature sensing process that prevents germination too close to the surface (Saatkamp et al. 2011), allowing the detection of safe locations and short‐term regeneration windows for seedling establishment (Thompson and Grime 1983; Pons 2000; Finch‐Savage and Leubner‐Metzger 2006; Carta et al. 2017).

Furthermore, warm stratification and scarification promote germination of *Mediterranean* species, which is particularly evident in the rosids, that in our study includes large number of Fabaceae (Figure S1), reflecting the strong overall positive effect of scarification in this hard‐seeded family (Figure S3). On the contrary, cold‐wet stratification, a common dormancy release mechanism in seeds from wet temperate biomes, was detrimental for germination in *Mediterranean* species (Skordilis and Thanos 1995; Luna et al. 2008). Since our results showed that *Mediterranean* species exhibit heavier seeds, compared to *habitat specialists*, it is not surprising to find a negative interactive effect of seed mass with temperature, alternating temperatures and light. This indicates that heavier seeds can germinate at cooler temperatures and greater depths, avoiding harsh surface conditions but still being able to growth and reach the surface because of their nutrient reserves (Pons 2000; Carta et al. 2017) in line with the Mediterranean seed germination syndrome. The positive effect of fire on the germination response highlights that heat stimulates the germination of species with water‐impermeable seeds, mostly Cistaceae and Fabaceae (González‐Rabanal and Casal 1995; Herranz, Ferrandis, and Martínez‐Sánchez 1998, 1999; Paula and Pausas 2008; Luna et al. 2023), that, as mentioned before, are particularly representative of Mediterranean habitat and phylogenetic diversity of our data set (Figure S2; Chytrý et al. 2020; Cruz‐Tejada et al. 2024). Overall, these germination responses align the regeneration niche phenology with the beginning of autumn/winter seasons, reflecting evolutionary pressures to avoid hot and drought stress and maximise recruitment during the season of highest water availability (Thanos, Kadis, and Skarou 1995; Doussi and Thanos 2002; Picciau et al. 2019; Carta et al. 2022; Mattana et al. 2022).

The contrasting germination responses between *Mediterranean* lowland species and *habitat specialists*, indicate that even within one main macroclimatic region (i.e., the Mediterranean Basin), plant species can exhibit distinct germination strategies due to local environmental pressures. For example, it has been reported (Picciau et al. 2019) that Mediterranean mountain species exhibit germination responses closer to species from temperate regions, positioning theme in between the typical Mediterranean germination syndrome and the alpine germination syndrome based on warm‐cued germination after cold stratification (Giménez‐Benavides, Escudero, and Pérez‐García 2005; Fernández‐Pascual et al. 2021), as showed by the *habitat specialists*. In addition, species occurring in wetlands deviate from the Mediterranean germination syndrome, reflecting responses to particular local conditions including water table fluctuations and light availability (Carta 2016; Rosbakh, Phartyal, and Poschlod 2020). On the contrary, despite not being solely influenced by macroclimatic conditions, coastal species show negative responses to light (Thanos et al. 1991; Carta et al. 2017) because germination on the surface of sand might present a hazard for newly germinated seedlings, due to rapid evaporation and large temperature fluctuations (De Vitis et al. 2014; Del Vecchio et al. 2020).

### 4.2 Future Directions and Study Limitations

Our results, and the data on which they are based, provide a solid basis for modelling the seed germination conditions under global warming scenarios (Gremer et al. 2020; Filipe et al. 2023; Mattana et al. 2023; Price et al. 2024). Additionally, by explicitly linking our results to the EUNIS classification of habitats, we provide valuable information to inform policies and support ecological restoration of Mediterranean ecosystems (Ladouceur et al. 2018; Arranz et al. 2024). However, to properly understand plant responses at finer scales (e.g., at the habitat level), we require a larger amount of data to achieve sufficient representativeness of both macro‐ and microenvironmental gradients that can sustain robust analyses. For example, the number of species and experiments tested under fire conditions was limited (666 records, 46 species; Figure S2), and therefore our understanding of the role of fire (including smoke, Moreira et al. 2010) in plant recruitment from seeds in the Mediterranean Basin is constrained by the availability of experiments specifically designed to investigate the effect of this variable. Moreover, not enough data on germination rate (i.e., germination speed) were available to include this germination parameter, which is considered an additional aspect of the seed germination niche in the Mediterranean (Doussi and Thanos 2002). Our results regarding the general pattern of seed germination in the Mediterranean are dependent on our selection of study species which, despite being chosen to maximise the phylogenetic and ecological representativeness of the data set, can and will be improved by future efforts to generate and collect germination data for an increasing number of species. Indeed, we expect that these data and other already published germination data for a larger number of species will become available over the next years with the help of initiatives such as MedGermDB (Cruz‐Tejada et al. 2024) and SeedArc (Fernández‐Pascual et al. 2023), allowing us to extend our understanding of Mediterranean seed regeneration across the different world floras that have adapted to Mediterranean climates.

## 5 Conclusions

This study demonstrates how phylogenetically distant species exhibit similar germination responses supporting the Mediterranean seed germination syndrome hypothesis and suggesting an evolutionary convergence of the germination niche mediated by pressures at the macroclimatic level. However, this syndrome is not exhibited by all Mediterranean species, rather, the germination niche of habitat specialists deviates from it. This suggests habitat‐specific evolutionary pressures overriding the Mediterranean syndrome, further indicating the need to account for the interplay between macroclimatic and habitat conditions, when disentangling the role of environmental cues shaping the macroecology of regeneration strategies.

## Author Contributions

**Diana María Cruz‐Tejada:** data curation (supporting), formal analysis (equal), supervision (equal), writing – original draft (equal), writing – review and editing (equal). **Efisio Mattana:** supervision (equal), writing – review and editing (equal). **Sergey Rosbakh:** data curation (supporting), writing – review and editing (equal). **Eduardo Fernández‐Pascual:** data curation (supporting), supervision (equal), writing – review and editing (equal). **Angelino Carta:** data curation (supporting), formal analysis (equal), supervision (equal), writing – review and editing (equal).

## Conflicts of Interest

The authors declare no conflicts of interest.

## Supporting information


Appendix S1.

Data S1.

## Data Availability

The original data and R code for the analysis are attached as Appendix S1 and Data S1 (csv and pdf files) and will be accessed at the GitHub repository https://github.com/DianaCruzTejada/MediterraneanSyndrome. A version of record of the repository can be found at https://doi.org/10.5281/zenodo.13974572.
