# Supplementary material for: Macroclimatic Convergence and Habitat Specialisation Shape the Mediterranean Seed Germination Syndrome
Source: Ecol Evol. 2024 Nov 7;14(11):e70527. doi: 10.1002/ece3.70527 (PMC11542993; doi:10.1002/ece3.70527)
Supplement: Supplementary file 1 — Appendix S1. [file ECE3-14-e70527-s001.pdf]

Appendices to the paper

Cruz-Tejada, D.M. et al. Macroclimatic convergence and habitat specialization shape the Mediterranean seed germination syndrome

Table S1. EUNIS habitats (Chytrý et al., 2020) included in the dataset with germination data and their attribution to the groups used in the analyses (Lowland *Mediterranean* and local *habitat-specialists*).

Table S2. Summary of phylogenetic mixed models with Bayesian estimation (MCMCglmms) examining interactive effect of experimental cues and the species group (*Mediterranean* or local *habitat-specialists*) on final seed germination proportion across the full dataset. Fixed and random effects (±95% CI) are presented. Significant effects are in bold. On average, models were the results of 4000 samples.

Table S3. Summary of phylogenetic mixed models with Bayesian estimation (MCMCglmms) examining interactive effect of experimental cues and the species group (*Mediterranean* or local *habitat-specialists*) on final seed germination proportion across the angiosperm clades. Fixed and random effects (±95% CI) are presented. Significant effects are in bold. On average, models were the results of 4000 samples.

Figure S1. (a) Dataset composition by clade grouped by number of species, seed lots, and germination tests. The numbers inside the bars indicate the total quantities for each category. (b) Phylogenetic tree at family level showing the number of species, germination experiments (test number) and *Mediterranean* species proportion (Med. Species proportion) per family included in the dataset.

Figure S2. Dataset contents: number and percentage of records for each experimental cue. The numbers correspond to the quantity of germination experiments included in the dataset for each experimental cue. In parenthesis there is the corresponded percentage. Not all experimental cues are exclusive.

Figure S3. Effect of the experimental cues on final germination proportions according to the MCMC meta-analysis of primary data across each major angiosperm clade. Dots indicate the posterior mean of the effect size, and whiskers its 95% credible interval. The line of zero effect is shown. When the credible intervals overlap with the zero-effect line, the effect is not significant. The figure shows the effect of experimental cue on seed germination in *Mediterranean* lowland species (orange), in local *habitat-specialists* pants (turkish blue) and the main effect (dark). The dark green point indicates the interactive effect of seed mass. A negative effect indicates that the response to the experimental cue (e.g. temperature) decreases when the mean temperature (environmental cue) is high.

Figure S4. Visualization of the seed germination niche of the Mediterranean plants through a Multiple Factor Analysis (MFA) coloured by major clade. Each dot is a species. Solid arrows correspond to loadings of the seed germination experimental conditions used to construct the ordination. Dashed arrows correspond to supplementary variables, not used to construct the ordination.

R codes. Codes for the analyses used in the manuscript.

Table S1. EUNIS habitats (Chytrý et al., 2020) included in the dataset with germination data and their attribution to the groups used in the analyses (Lowland *Mediterranean* and local *habitat-specialists*).

EUNIS habitat level 3	Groups	MacroHabitat
Acidophilous Quercus forest	*habitat-specialists*	forest
Alkaline, calcareous, carbonate-rich small-sedge spring fen	*habitat-specialists*	wetlands
Alnus cordata forest	*habitat-specialists*	forest
Alnus glutinosa-Alnus incana forest on riparian and mineral soils	*habitat-specialists*	forest
Alpine and subalpine calcareous grassland of the Balkans and Apennines	*habitat-specialists*	grassland
Alpine and subalpine ericoid heath	*habitat-specialists*	schrubland
Alpine and subalpine Juniperus scrub	*habitat-specialists*	schrubland
Annual anthropogenic herbaceous vegetation	*habitat-specialists*	Man-made
Arable land with unmixed crops grown by low-intensity agricultural methods	*habitat-specialists*	Man-made
Aralo-Caspian semi-desert	*habitat-specialists*	schrubland
Arctic-alpine calcareous grassland	*habitat-specialists*	grassland
Arctic-alpine rich fen	*habitat-specialists*	wetlands
Atlantic and Baltic broad-leaved coastal dune forest	*habitat-specialists*	coastal
Atlantic and Baltic coastal dune grassland (grey dune)	*habitat-specialists*	coastal
Atlantic and Baltic coastal dune scrub	*habitat-specialists*	coastal
Atlantic and Baltic coastal Empetrum heath	*habitat-specialists*	coastal
Atlantic and Baltic moist and wet dune slack	*habitat-specialists*	coastal
Atlantic and Baltic rocky sea cliff and shore	*habitat-specialists*	coastal
Atlantic and Baltic shifting coastal dune	*habitat-specialists*	coastal
Atlantic and Baltic soft sea cliff	*habitat-specialists*	coastal
Atlantic coastal Calluna and Ulex heath	*habitat-specialists*	coastal
Atlantic, Baltic and Arctic coastal shingle beach	*habitat-specialists*	coastal
Atlantic, Baltic and Arctic sand beach	*habitat-specialists*	coastal
Azorean open, dry, acid to neutral grassland	*habitat-specialists*	grassland
Balkan-Anatolian submontane genistoid scrub	*habitat-specialists*	schrubland
Balkan and Anatolian oromediterranean dry grassland	*habitat-specialists*	grassland
Baltic coniferous coastal dune forest	*habitat-specialists*	coastal
Bare tilled, fallow or recently abandoned arable land	*habitat-specialists*	Man-made
Black Sea broad-leaved coastal dune forest	*habitat-specialists*	coastal
Black Sea coastal dune grassland (grey dune)	*habitat-specialists*	coastal
Blanket bog	*habitat-specialists*	wetlands
Boreal and Arctic acidophilous alpine grassland	*habitat-specialists*	grassland
Broadleaved deciduous plantation of non site-native trees	*habitat-specialists*	forest
Broadleaved evergreen plantation of non site-native trees	*habitat-specialists*	forest
Broadleaved mire forest on acid peat	*habitat-specialists*	forest
Broadleaved swamp forest on non-acid peat	*habitat-specialists*	forest
Calcareous quaking mire	*habitat-specialists*	wetlands
Canarian xerophytic scrub	*habitat-specialists*	schrubland
Carpathian travertine fen with halophytes	*habitat-specialists*	wetlands
Carpinus and Quercus mesic deciduous forest	*habitat-specialists*	forest
Central Mediterranean mountain hedgehog-heath	*habitat-specialists*	schrubland
Coniferous plantation of non site-native trees	*habitat-specialists*	forest
Continental dry grassland (true steppe)	*habitat-specialists*	grassland
Continental dry rocky steppic grassland and dwarf scrub on chalk outcrops	*habitat-specialists*	grassland

Continental inland salt steppe	*habitat-specialists*	grassland
Continental subsaline alluvial pasture and meadow	*habitat-specialists*	grassland
Corylus avellana scrub	*habitat-specialists*	schrubland
Cryptogam- and annual-dominated vegetation on calcareous and ultramafic rock outcrops	*habitat-specialists*	grassland
Cryptogam- and annual-dominated vegetation on siliceous rock outcrops	*habitat-specialists*	grassland
Cyrno-Sardean oromediterranean siliceous dry grassland	*habitat-specialists*	grassland
Dark taiga	*habitat-specialists*	forest
Desert steppe	*habitat-specialists*	grassland
Dry heath	*habitat-specialists*	schrubland
Dry Mediterranean land with unpalatable non-vernal herbaceous vegetation	*lowland Mediterranean*	Man-made
Dry perennial anthropogenic herbaceous vegetation	*habitat-specialists*	Man-made
Dry steppic submediterranean pasture of the Amphi-Adriatic region	*habitat-specialists*	grassland
Eastern garrigue	*lowland Mediterranean*	schrubland
Eastern Mediterranean mountain hedgehog-heath	*habitat-specialists*	schrubland
Eastern Mediterranean spiny heath (phrygana)	*lowland Mediterranean*	schrubland
Extremely rich moss-sedge fen	*habitat-specialists*	wetlands
Fagus forest on acid soils	*habitat-specialists*	forest
Fagus forest on non-acid soils	*habitat-specialists*	forest
Forest fringe of acidic nutrient-poor soils	*habitat-specialists*	grassland
Heavy-metal dry grassland of the Balkans	*habitat-specialists*	grassland
Heavy-metal grassland in Western and Central Europe	*habitat-specialists*	grassland
Herbaceous forest clearing vegetation	*habitat-specialists*	grassland
Iberian oromediterranean basiphilous dry grassland	*habitat-specialists*	grassland
Iberian oromediterranean siliceous dry grassland	*habitat-specialists*	grassland
Iberian summer pasture (vallicar)	*habitat-specialists*	grassland
Ilex aquifolium forest	*habitat-specialists*	forest
Inland saline or brackish helophyte bed	*habitat-specialists*	wetlands
Inland sanddrift and dune with siliceous grassland	*habitat-specialists*	grassland
Intensive unmixed crops	*habitat-specialists*	Man-made
Intermediate fen and soft-water spring mire	*habitat-specialists*	wetlands
Inundated or inundatable cropland, including rice fields	*habitat-specialists*	Man-made
Larix light taiga	*habitat-specialists*	forest
Low and medium altitude hay meadow	*habitat-specialists*	grassland
Low steppic scrub	*habitat-specialists*	schrubland
Lowland moist or wet tall-herb and fern fringe	*habitat-specialists*	grassland
Lowland to montane temperate and submediterranean genistoid scrub	*habitat-specialists*	schrubland
Lowland to montane temperate and submediterranean Juniperus scrub	*habitat-specialists*	schrubland
Lowland to montane, dry to mesic grassland usually dominated by Nardus stricta	*habitat-specialists*	grassland
Macaronesian coastal dune scrub	*habitat-specialists*	coastal
Macaronesian garrigue	*habitat-specialists*	schrubland
Macaronesian heath	*habitat-specialists*	schrubland
Macaronesian heathy forest	*habitat-specialists*	forest
Macaronesian Juniperus forest	*habitat-specialists*	forest
Macaronesian laurophyllous forest	*habitat-specialists*	forest
Macaronesian rocky sea cliff and shore	*habitat-specialists*	coastal
Macaronesian thermophilous forest fringe	*habitat-specialists*	grassland
Madeiran oromediterranean siliceous dry grassland	*habitat-specialists*	grassland

Madeiran xerophytic scrub	*habitat-specialists*	schrubland
Mainland laurophyllous forest	*habitat-specialists*	forest
Mediterranean and Balkan subalpine Pinus heldreichii-Pinus peuce forest	*habitat-specialists*	forest
Mediterranean and Black Sea coastal dune scrub	*habitat-specialists*	coastal
Mediterranean and Black Sea coastal shingle beach	*habitat-specialists*	coastal
Mediterranean and Black Sea moist and wet dune slack	*habitat-specialists*	coastal
Mediterranean and Black Sea rocky sea cliff and shore	*habitat-specialists*	coastal
Mediterranean and Black Sea sand beach	*habitat-specialists*	coastal
Mediterranean and Black Sea soft sea cliff	*habitat-specialists*	coastal
Mediterranean and Macaronesian coastal dune grassland (grey dune)	*habitat-specialists*	coastal
Mediterranean and Macaronesian riparian forest	*habitat-specialists*	forest
Mediterranean annual-rich dry grassland	*lowland Mediterranean*	grassland
Mediterranean closely grazed dry grassland	*lowland Mediterranean*	grassland
Mediterranean coniferous coastal dune forest	*habitat-specialists*	coastal
Mediterranean Cupressaceae forest	*lowland Mediterranean*	forest
Mediterranean evergreen Quercus forest	*lowland Mediterranean*	forest
Mediterranean gypsum scrub	*lowland Mediterranean*	schrubland
Mediterranean halo-nitrophilous scrub	*lowland Mediterranean*	schrubland
Mediterranean inland salt steppe	*lowland Mediterranean*	grassland
Mediterranean lowland to submontane Pinus forest	*habitat-specialists*	forest
Mediterranean maquis and arborescent matorral	*lowland Mediterranean*	schrubland
Mediterranean montane Cedrus forest	*habitat-specialists*	forest
Mediterranean montane Pinus sylvestris-Pinus nigra forest	*habitat-specialists*	forest
Mediterranean mountain Abies forest	*habitat-specialists*	forest
Mediterranean riparian scrub	*habitat-specialists*	schrubland
Mediterranean short moist grassland of lowlands	*lowland Mediterranean*	grassland
Mediterranean short moist grassland of mountains	*habitat-specialists*	grassland
Mediterranean subnitrophilous annual grassland	*lowland Mediterranean*	Man-made
Mediterranean tall humid inland grassland	*lowland Mediterranean*	grassland
Mediterranean tall perennial dry grassland	*lowland Mediterranean*	grassland
Mediterranean thermophilous deciduous forest	*lowland Mediterranean*	forest
Mediterranean to Atlantic open, dry, acid and neutral grassland	*habitat-specialists*	grassland
Mediterranean, Macaronesian and Black Sea shifting coastal dune	*habitat-specialists*	coastal
Mesic perennial anthropogenic herbaceous vegetation	*habitat-specialists*	Man-made
Mesic permanent pasture of lowlands and mountains	*habitat-specialists*	grassland
Mixed crops of market gardens and horticulture	*habitat-specialists*	Man-made
Moist or wet mesotrophic to eutrophic hay meadow	*habitat-specialists*	grassland
Moist or wet mesotrophic to eutrophic pasture	*habitat-specialists*	grassland
Montane to subalpine moist or wet tall-herb and fern fringe	*habitat-specialists*	grassland
Moss and lichen tundra	*habitat-specialists*	schrubland
Mountain hay meadow	*habitat-specialists*	grassland
Non-calcareous quaking mire	*habitat-specialists*	wetlands
Oceanic to subcontinental inland sand grassland on dry acid and neutral soils	*habitat-specialists*	grassland
Oceanic valley mire	*habitat-specialists*	wetlands
Olea europaea-Ceratonia siliqua forest	*lowland Mediterranean*	forest
Open Iberian supramediterranean dry acid and neutral grassland	*habitat-specialists*	grassland
Pannonian and Pontic sandy steppe	*habitat-specialists*	grassland

Perennial rocky calcareous grassland of subatlantic-submediterranean Europe	*habitat-specialists*	grassland
Perennial rocky grassland of Central and South-Eastern Europe	*habitat-specialists*	grassland
Perennial rocky grassland of the Italian Peninsula	*habitat-specialists*	grassland
Phoenix canariensis vegetation	*habitat-specialists*	forest
Phoenix theophrasti vegetation	*habitat-specialists*	forest
Picea mire forest	*habitat-specialists*	forest
Pinus and Larix mire forest	*habitat-specialists*	forest
Pinus canariensis forest	*habitat-specialists*	forest
Pinus sylvestris light taiga	*habitat-specialists*	forest
Poor fen	*habitat-specialists*	wetlands
Pteridium aquilinum vegetation	*habitat-specialists*	grassland
Raised bog	*habitat-specialists*	wetlands
Ravine forest	*habitat-specialists*	forest
Relict mire of Mediterranean mountains	*habitat-specialists*	wetlands
Salix fen scrub	*habitat-specialists*	schrubland
Semi-desert riparian scrub	*habitat-specialists*	schrubland
Semi-desert salt pan	*habitat-specialists*	grassland
Semi-desert sand dune with sparse scrub	*habitat-specialists*	schrubland
Semi-dry perennial calcareous grassland (meadow steppe)	*habitat-specialists*	grassland
Shrub tundra	*habitat-specialists*	schrubland
Small-helophyte bed	*habitat-specialists*	wetlands
Snow-bed vegetation	*habitat-specialists*	grassland
Southern European mountain Betula and Populus tremula forest on mineral soils	*habitat-specialists*	forest
Spartium junceum scrub	*habitat-specialists*	schrubland
Subalpine and subarctic deciduous scrub	*habitat-specialists*	schrubland
Subalpine genistoid scrub of the Amphi-Adriatic region	*habitat-specialists*	schrubland
Subalpine Pinus mugo scrub	*habitat-specialists*	schrubland
Subarctic and alpine dwarf Salix scrub	*habitat-specialists*	schrubland
Submediterranean moist meadow	*habitat-specialists*	grassland
Submediterranean pseudomaquis	*habitat-specialists*	schrubland
Tall-helophyte bed	*habitat-specialists*	wetlands
Tall-sedge base-rich fen	*habitat-specialists*	wetlands
Tall-sedge bed	*habitat-specialists*	wetlands
Taxus baccata forest	*habitat-specialists*	forest
Temperate acidophilous alpine grassland	*habitat-specialists*	grassland
Temperate and boreal moist or wet oligotrophic grassland	*habitat-specialists*	grassland
Temperate and boreal mountain Betula and Populus tremula forest on mineral soils	*habitat-specialists*	forest
Temperate and submediterranean montane Pinus sylvestris-Pinus nigra forest	*habitat-specialists*	forest
Temperate and submediterranean thermophilous deciduous forest	*habitat-specialists*	forest
Temperate and submediterranean thorn scrub	*habitat-specialists*	schrubland
Temperate continental Pinus sylvestris forest	*habitat-specialists*	forest
Temperate forest clearing scrub	*habitat-specialists*	schrubland
Temperate hardwood riparian forest	*habitat-specialists*	forest
Temperate inland salt marsh	*habitat-specialists*	grassland
Temperate mountain Abies forest	*habitat-specialists*	forest
Temperate mountain Picea forest	*habitat-specialists*	forest
Temperate riparian scrub	*habitat-specialists*	schrubland

Temperate Rubus scrub	*habitat-specialists*	schrubland
Temperate Salix and Populus riparian forest	*habitat-specialists*	forest
Temperate subalpine Larix, Pinus cembra and Pinus uncinata forest	*habitat-specialists*	forest
Thermomediterranean arid scrub	*lowland Mediterranean*	schrubland
Thermophilous forest fringe of base-rich soils	*habitat-specialists*	grassland
Trampled mesophilous grassland with annuals	*habitat-specialists*	Man-made
Trampled xeric grassland with annuals	*habitat-specialists*	Man-made
Western acidophilous garrigue	*habitat-specialists*	schrubland
Western basiphilous garrigue	*habitat-specialists*	schrubland
Western Mediterranean mountain hedgehog-heath	*habitat-specialists*	schrubland
Western Mediterranean spiny heath	*lowland Mediterranean*	schrubland
Wet heath	*habitat-specialists*	schrubland

Table S2. Summary of phylogenetic mixed models with Bayesian estimation (MCMCglmms) examining interactive effect of experimental cues and the species group (lowland *Mediterranean* or local *habitat-specialists*) on final seed germination proportion across the full dataset. Fixed and random effects (±95% CI) are presented. Significant effects are in bold. On average, models were the results of 4000 samples.

Model	Fixed effects			Random effects					
	**Post.mean**	**pMCMC**	**CI.95**	**Phylogeny**	**Species**	**Id test**	**doi**	**Seedlot**	**Substrate**
**Temperature**									
Main effect	0.156	0	(0.105;0.205)	2.27(1.214;3.448)	1.086(0.7;1.501)	3.142(2.658;3.57)	2.545(1.791;3.346)	1.456(1.252;1.677)	0.227(0;0.907)
Mediterranean	-0.16	0	(-0.211;-0.11)						
Habitat-specialists	0.16	0	(0.109;0.208)	2.272(1.186;3.503)	1.099(0.717;1.508)	3.142(2.649;3.558)	2.556(1.834;3.368)	1.457(1.251;1.678)	0.281(0;1.065)
Seed mass	-0.08	0	(-0.104;-0.056)	2.298(1.197;3.459)	1.105(0.714;1.532)	3.167(2.699;3.581)	2.457(1.739;3.235)	1.438(1.224;1.659)	0.286(0;0.98)
**Alternating**									
Main effect	0.31	0	(0.231;0.386)	2.424(1.281;3.717)	1.107(0.69;1.51)	3.118(2.632;3.571)	2.664(1.835;3.429)	1.464(1.261;1.677)	0.21(0;0.847)
Mediterranean	-0.068	0.052	(-0.135;0.001)						
Habitat-specialists	0.069	0.045	(0.004;0.142)	2.401(1.28;3.685)	1.112(0.733;1.535)	3.128(2.668;3.565)	2.652(1.84;3.441)	1.468(1.258;1.686)	0.293(0;1.102)
Seed mass	-0.006	0.769	(-0.044;0.028)	2.264(1.18;3.435)	1.101(0.721;1.533)	3.197(2.724;3.615)	2.481(1.782;3.291)	1.429(1.216;1.643)	0.332(0;1.159)
**Light**									
Main effect	0.304	0	(0.226;0.379)	2.182(1.138;3.416)	1.106(0.71;1.517)	3.167(2.703;3.613)	2.54(1.775;3.365)	1.487(1.271;1.706)	0.27(0;1.029)
Mediterranean	-0.088	0.016	(-0.156;-0.018)						
Habitat-specialists	0.088	0.018	(0.019;0.16)	2.158(1.076;3.286)	1.103(0.704;1.506)	3.164(2.714;3.592)	2.561(1.739;3.368)	1.49(1.277;1.713)	0.391(0;1.534)
Seed mass	-0.088	0	(-0.123;-0.053)	2.26(1.205;3.531)	1.128(0.731;1.54)	3.2(2.747;3.638)	2.438(1.692;3.298)	1.479(1.269;1.698)	0.207(0;0.831)
**Cold str**									
Main effect	0.299	0	(0.236;0.37)	2.502(1.323;3.831)	1.198(0.799;1.655)	3.082(2.639;3.534)	2.677(1.904;3.538)	1.485(1.267;1.697)	0.35(0;1.09)
Mediterranean	-0.202	0	(-0.265;-0.134)						
Habitat-specialists	0.202	0	(0.138;0.267)	2.529(1.36;3.917)	1.191(0.77;1.636)	3.083(2.649;3.505)	2.694(1.91;3.533)	1.48(1.278;1.695)	0.329(0;0.975)
Seed mass	0.087	0	(0.057;0.117)	2.463(1.311;3.843)	1.084(0.676;1.485)	3.158(2.68;3.587)	2.669(1.893;3.478)	1.456(1.237;1.659)	0.306(0;1.169)
**Warm str**									
Main effect	-0.112	0	(-0.175;-0.047)	2.161(1.092;3.291)	1.09(0.722;1.503)	3.032(2.537;3.446)	2.47(1.769;3.234)	1.46(1.244;1.669)	0.404(0;1.329)
Mediterranean	0.459	0	(0.393;0.529)						
Habitat-specialists	-0.459	0	(-0.523;-0.389)	2.099(1.088;3.183)	1.106(0.73;1.522)	3.03(2.559;3.461)	2.462(1.682;3.2)	1.458(1.261;1.673)	0.331(0;1.406)
Seed mass	0.005	0.786	(-0.028;0.038)	2.231(1.209;3.448)	1.097(0.679;1.472)	3.188(2.699;3.615)	2.476(1.761;3.254)	1.43(1.236;1.663)	0.28(0;1.046)
**Scarification**									
Main effect	0.693	0	(0.585;0.795)	2.369(1.207;3.596)	1.202(0.797;1.627)	2.983(2.515;3.434)	2.657(1.902;3.456)	1.442(1.23;1.643)	0.222(0;0.872)
Mediterranean	0.115	0.013	(0.022;0.204)						
Habitat-specialists	-0.114	0.011	(-0.202;-0.022)	2.372(1.187;3.608)	1.209(0.77;1.629)	2.986(2.523;3.386)	2.618(1.88;3.412)	1.441(1.236;1.65)	0.231(0;0.755)
Seed mass	0.166	0	(0.128;0.207)	2.499(1.401;3.851)	1.089(0.69;1.496)	3.117(2.655;3.578)	2.575(1.818;3.349)	1.471(1.257;1.686)	0.219(0;0.865)
**Fire**									
Main effect	0.15	0.002	(0.054;0.253)	2.253(1.154;3.375)	1.094(0.697;1.481)	3.161(2.674;3.584)	2.617(1.908;3.498)	1.439(1.234;1.657)	0.253(0;0.944)
Mediterranean	0.003	0.935	(-0.087;0.09)						
Habitat-specialists	-0.003	0.965	(-0.09;0.089)	2.274(1.166;3.463)	1.081(0.712;1.522)	3.158(2.681;3.607)	2.593(1.864;3.445)	1.439(1.234;1.662)	0.23(0;0.817)
Seed mass	-0.064	0.001	(-0.102;-0.027)	2.208(1.072;3.315)	1.088(0.697;1.486)	3.185(2.732;3.615)	2.447(1.75;3.255)	1.425(1.219;1.636)	0.231(0;0.913)

Table S3. Summary of phylogenetic mixed models with Bayesian estimation (MCMCglmms) examining interactive effect of experimental cues and the species group (lowland *Mediterranean* or local *habitat-specialists*) on final seed germination proportion across the angiosperm clades. Fixed and random effects (±95% CI) are presented. Significant effects are in bold. On average, models were the results of 4000 samples.

Model	Fixed effects			Random effects					
	**Post.mean**	**pMCMC**	**CI.95**	**Phylogeny**	**Species**	**Id test**	**doi**	**Seedlot**	**Substrate**
				**MONOCOTS**					
**Temperature**									
Main effect	0.401	0	(0.324;0.485)	3.346(0.948;6.677)	1.051(0.479;1.697)	1.396(0.895;1.909)	2.259(1.012;3.64)	1.227(0.935;1.517)	1.496(0;5.765)
Mediterranean	0.069	0.097	(-0.015;0.149)						
Habitat-specialists	-0.067	0.106	(-0.147;0.013)	3.244(0.91;6.461)	1.064(0.468;1.706)	1.396(0.866;1.852)	2.264(1.015;3.646)	1.223(0.942;1.526)	1.545(0;6.241)
Seed mass	-0.168	0	(-0.228;-0.101)	3.129(0.604;6.044)	1.172(0.571;1.846)	1.47(0.951;1.934)	2.311(1.115;3.645)	1.178(0.912;1.479)	1.473(0;6.142)
**Alternating**									
Main effect	0.13	0.036	(0.006;0.248)	3.171(0.749;6.076)	1.128(0.549;1.787)	1.498(0.983;1.952)	2.234(1.004;3.541)	1.189(0.892;1.471)	1.683(0;5.999)
Mediterranean	0.01	0.877	(-0.096;0.117)						
Habitat-specialists	-0.01	0.856	(-0.117;0.103)	3.188(0.907;6.237)	1.118(0.527;1.773)	1.517(1.038;1.984)	2.197(1.018;3.493)	1.19(0.916;1.487)	1.775(0;5.509)
Seed mass	-0.178	0	(-0.247;-0.111)	2.913(0.574;5.809)	1.181(0.573;1.862)	1.472(0.946;1.923)	1.955(0.88;3.223)	1.2(0.911;1.496)	1.614(0;5.962)
**Light**									
Main effect	0.114	0.013	(0.025;0.208)	2.859(0.479;5.567)	1.105(0.518;1.723)	1.49(0.99;1.996)	2.32(1.115;3.814)	1.192(0.907;1.48)	1.861(0;7.371)
Mediterranean	0.029	0.546	(-0.063;0.123)						
Habitat-specialists	-0.031	0.504	(-0.123;0.061)	2.746(0.631;5.4)	1.114(0.581;1.805)	1.497(0.955;1.943)	2.286(1.01;3.679)	1.193(0.905;1.483)	1.783(0;6.469)
Seed mass	-0.1	0	(-0.151;-0.054)	2.993(0.725;5.833)	1.097(0.539;1.769)	1.496(0.972;1.961)	2.306(1.053;3.767)	1.166(0.886;1.453)	2.113(0;8.243)
**Cold str**									
Main effect	0.173	0.01	(0.039;0.305)	3.168(0.593;5.922)	1.168(0.553;1.842)	1.494(0.965;1.928)	2.385(1.064;3.807)	1.176(0.895;1.458)	2.395(0;7.558)
Mediterranean	-0.023	0.717	(-0.145;0.103)						
Habitat-specialists	0.025	0.708	(-0.1;0.152)	3.166(0.782;6.145)	1.147(0.559;1.83)	1.479(0.936;1.95)	2.35(1.103;3.74)	1.181(0.918;1.482)	1.657(0;6.502)
Seed mass	-0.092	0.02	(-0.166;-0.015)	3.11(0.762;6.061)	1.079(0.473;1.715)	1.519(0.983;1.994)	2.166(1.036;3.612)	1.139(0.871;1.429)	1.784(0;6.426)
**Warm str**									
Main effect	-0.112	0.092	(-0.241;0.02)	2.993(0.67;5.89)	1.085(0.499;1.71)	1.536(1.047;2.022)	2.205(1.052;3.564)	1.174(0.859;1.447)	1.924(0;6.411)
Mediterranean	-0.015	0.827	(-0.142;0.118)						
Habitat-specialists	0.015	0.831	(-0.112;0.146)	2.993(0.586;5.786)	1.088(0.539;1.735)	1.519(0.997;2)	2.208(1.01;3.527)	1.17(0.89;1.457)	1.864(0;6.702)
Seed mass	-0.086	0.038	(-0.171;-0.009)	2.893(0.635;5.72)	1.082(0.504;1.688)	1.523(1.024;2.007)	2.184(1.033;3.469)	1.157(0.893;1.461)	1.595(0;5.938)
**Scarification**									
Main effect	0.034	0.472	(-0.053;0.13)	3.164(0.766;6.11)	1.145(0.559;1.84)	1.518(0.997;1.982)	2.178(1.073;3.586)	1.168(0.903;1.49)	1.795(0;6.936)
Mediterranean	-0.053	0.252	(-0.143;0.043)						
Habitat-specialists	0.055	0.233	(-0.036;0.145)	3.198(0.73;6.17)	1.144(0.573;1.833)	1.512(1.015;2.005)	2.173(1.008;3.44)	1.173(0.898;1.47)	1.801(0;6.36)
Seed mass	-0.089	0.004	(-0.146;-0.029)	2.967(0.671;5.732)	1.066(0.475;1.68)	1.522(1.008;1.995)	2.208(1.018;3.548)	1.155(0.883;1.455)	2.077(0;6.855)
**Fire**									
Main effect	2.009	0.314	(-1.91;6.038)	2.962(0.639;5.806)	1.132(0.558;1.786)	1.524(1.009;2.002)	2.264(1.014;3.612)	1.165(0.886;1.47)	1.654(0;5.864)

Mediterranean	-2.118	0.297	(-6.269;1.859)						
Habitat-specialists	2.135	0.293	(-1.794;6.286)	2.91(0.743;5.909)	1.13(0.514;1.756)	1.525(1.013;1.998)	2.234(1.097;3.666)	1.161(0.875;1.461)	1.833(0;6.807)
Seed mass	-0.038	0.126	(-0.088;0.014)	2.993(0.66;5.739)	1.118(0.541;1.789)	1.53(0.989;1.953)	2.187(1.004;3.464)	1.161(0.896;1.46)	1.77(0;6.14)
				**ROSIDS**					
**Temperature**									
Main effect	-0.146	0	(-0.227;-0.055)	2.937(0.707;5.515)	0.938(0;1.812)	3.284(2.773;3.775)	2.772(1.723;3.91)	0.878(0.575;1.154)	1.007(0;3.909)
Mediterranean	-0.093	0.032	(-0.182;-0.013)						
Habitat-specialists	0.092	0.028	(0.01;0.176)	3.01(0.823;5.831)	0.925(0;1.815)	3.279(2.727;3.782)	2.797(1.682;3.911)	0.878(0.608;1.19)	1.224(0;4.394)
Seed mass	-0.069	0	(-0.101;-0.04)	3.04(0.776;5.663)	0.941(0;1.832)	3.263(2.754;3.805)	2.692(1.623;3.779)	0.874(0.603;1.172)	1.43(0;5.467)
**Alternating**									
Main effect	0.22	0	(0.091;0.342)	2.825(0.673;5.369)	0.971(0.034;1.855)	3.254(2.706;3.742)	2.998(1.98;4.268)	0.878(0.604;1.19)	0.938(0;3.459)
Mediterranean	-0.2	0	(-0.307;-0.084)						
Habitat-specialists	0.2	0	(0.093;0.316)	2.993(0.858;5.702)	0.912(0;1.774)	3.243(2.709;3.747)	3.007(1.922;4.265)	0.891(0.597;1.189)	1.121(0;3.834)
Seed mass	0.037	0.099	(-0.006;0.083)	2.958(0.799;5.608)	0.94(0;1.83)	3.288(2.759;3.778)	2.889(1.787;4.03)	0.879(0.612;1.195)	1.099(0;4.113)
**Light**									
Main effect	0.115	0.014	(0.024;0.207)	2.797(0.646;5.558)	1.096(0.517;1.728)	1.495(0.992;1.972)	2.363(1.044;3.74)	1.195(0.905;1.479)	1.658(0;7.022)
Mediterranean	0.029	0.533	(-0.06;0.125)						
Habitat-specialists	-0.03	0.527	(-0.118;0.063)	2.822(0.652;5.747)	1.106(0.547;1.767)	1.488(0.952;1.95)	2.349(1.112;3.796)	1.194(0.91;1.491)	2.132(0;6.776)
Seed mass	-0.1	0	(-0.146;-0.052)	3.144(0.787;6.115)	1.08(0.501;1.711)	1.492(1.003;2.015)	2.303(1.161;3.759)	1.17(0.893;1.463)	2.164(0;8.939)
**Cold str**									
Main effect	0.31	0	(0.169;0.45)	3.567(1.078;6.778)	0.959(0;1.872)	2.921(2.434;3.396)	3.711(2.352;5.181)	0.986(0.704;1.304)	1.195(0;4.794)
Mediterranean	-0.458	0	(-0.582;-0.322)						
Habitat-specialists	0.459	0	(0.325;0.58)	3.596(1.082;6.833)	0.936(0;1.853)	2.915(2.413;3.442)	3.68(2.346;5.115)	0.989(0.694;1.292)	1.323(0;4.758)
Seed mass	0.175	0	(0.138;0.211)	4.706(1.519;8.024)	0.768(0;1.672)	3.053(2.517;3.538)	4.067(2.583;5.685)	0.942(0.658;1.254)	1.104(0;4.1)
**Warm str**									
Main effect	-0.182	0	(-0.291;-0.08)	2.727(0.692;5.343)	0.924(0;1.779)	3.179(2.665;3.703)	2.805(1.718;3.957)	0.926(0.633;1.218)	1.648(0;5.139)
Mediterranean	0.212	0	(0.121;0.316)						
Habitat-specialists	-0.213	0	(-0.314;-0.116)	2.736(0.651;5.375)	0.942(0;1.81)	3.191(2.635;3.661)	2.855(1.815;4.048)	0.923(0.635;1.222)	1.666(0;5.659)
Seed mass	-0.103	0	(-0.132;-0.074)	2.571(0.552;4.971)	0.882(0;1.702)	3.22(2.712;3.718)	2.658(1.675;3.776)	0.923(0.628;1.225)	1.259(0;4.644)
**Scarification**									
Main effect	1.274	0	(1.139;1.409)	2.841(0.295;5.706)	1.188(0.198;2.297)	2.569(2.039;3.017)	3.429(2.235;4.763)	0.766(0.537;1.028)	0.864(0;3.442)
Mediterranean	0.068	0.304	(-0.067;0.193)						
Habitat-specialists	-0.065	0.327	(-0.202;0.062)	2.911(0.501;5.924)	1.174(0.171;2.182)	2.569(2.061;3.04)	3.433(2.278;4.758)	0.768(0.545;1.027)	0.86(0;3.178)
Seed mass	0.251	0	(0.202;0.307)	3.856(1.344;7.032)	0.949(0;1.826)	3.065(2.532;3.555)	3.625(2.309;5.135)	0.896(0.616;1.21)	1.142(0;4.27)
**Fire**									
Main effect	0.349	0	(0.201;0.496)	2.785(0.742;5.353)	0.97(0;1.825)	3.201(2.706;3.718)	3.207(2.06;4.479)	0.894(0.62;1.207)	1.072(0;3.831)
Mediterranean	0.021	0.767	(-0.129;0.162)						
Habitat-specialists	-0.02	0.791	(-0.171;0.122)	2.781(0.663;5.365)	0.985(0;1.864)	3.197(2.695;3.691)	3.214(2.033;4.463)	0.889(0.604;1.19)	1.437(0;4.6)
Seed mass	-0.075	0.015	(-0.133;-0.017)	2.696(0.706;5.186)	0.968(0;1.833)	3.289(2.753;3.79)	2.767(1.722;3.936)	0.87(0.588;1.167)	1.323(0;4.764)

ASTERIDS

**Temperature**									
Main effect	0.327	0	(0.221;0.426)	2.574(0.421;5.328)	1.083(0.232;1.956)	3.598(3.038;4.174)	1.782(0.666;3.017)	2.44(1.738;3.181)	0.636(0;2.382)
Mediterranean	-0.392	0	(-0.495;-0.289)						
Habitat-specialists	0.392	0	(0.293;0.495)	2.581(0.361;5.156)	1.089(0.239;1.964)	3.6(3.02;4.168)	1.804(0.651;3.053)	2.425(1.692;3.134)	0.62(0;2.493)
Seed mass	-0.108	0	(-0.163;-0.053)	2.569(0.478;5.32)	1.049(0.18;1.949)	3.824(3.241;4.412)	1.787(0.659;3.053)	2.301(1.629;3.022)	0.61(0;2.602)
**Alternating**									
Main effect	0.656	0	(0.48;0.83)	2.483(0.194;5.097)	1.147(0.28;2.056)	3.682(3.09;4.279)	2.353(0.966;3.886)	2.448(1.707;3.139)	0.517(0;2.198)
Mediterranean	-0.216	0.004	(-0.361;-0.059)						
Habitat-specialists	0.214	0.004	(0.066;0.367)	2.619(0.486;5.44)	1.133(0.285;2.037)	3.66(3.067;4.22)	2.309(0.978;3.725)	2.439(1.779;3.24)	0.665(0;2.409)
Seed mass	0.028	0.5	(-0.057;0.11)	2.52(0.403;5.161)	1.043(0.252;1.96)	3.863(3.239;4.412)	1.794(0.695;3.058)	2.291(1.669;3.048)	0.701(0;2.413)
**Light**									
Main effect	0.647	0	(0.521;0.789)	2.503(0.44;5.202)	1.018(0.246;1.953)	3.518(2.979;4.101)	1.85(0.604;3.233)	2.64(1.877;3.376)	0.612(0;2.525)
Mediterranean	-0.396	0	(-0.527;-0.273)						
Habitat-specialists	0.397	0	(0.267;0.527)	2.504(0.342;5.16)	1.039(0.201;1.958)	3.499(2.923;4.056)	1.852(0.569;3.168)	2.631(1.927;3.438)	0.645(0;2.444)
Seed mass	-0.163	0	(-0.228;-0.102)	2.569(0.361;5.275)	1.106(0.233;2.086)	3.802(3.238;4.407)	1.577(0.572;2.747)	2.346(1.679;3.08)	0.62(0;2.521)
**Cold str**									
Main effect	0.163	0.028	(0.021;0.31)	2.744(0.394;5.769)	1.112(0.248;2.051)	3.85(3.224;4.397)	1.771(0.701;3.07)	2.31(1.617;3.036)	0.54(0;2.233)
Mediterranean	-0.12	0.088	(-0.262;0.01)						
Habitat-specialists	0.119	0.085	(-0.008;0.255)	2.703(0.397;5.563)	1.099(0.233;2.003)	3.836(3.294;4.415)	1.781(0.674;3.036)	2.304(1.574;2.994)	0.608(0;2.196)
Seed mass	-0.029	0.372	(-0.093;0.034)	2.614(0.511;5.495)	1.045(0.301;2.002)	3.853(3.249;4.453)	1.761(0.661;3.024)	2.301(1.645;2.989)	0.569(0;2.245)
**Warm str**									
Main effect	0.33	0	(0.168;0.507)	2.571(0.349;5.209)	1.106(0.264;2.039)	3.634(3.056;4.202)	1.766(0.579;3.05)	2.538(1.821;3.3)	0.584(0;1.874)
Mediterranean	0.172	0.024	(0.025;0.323)						
Habitat-specialists	-0.173	0.031	(-0.334;-0.02)	2.607(0.485;5.366)	1.085(0.21;1.965)	3.633(3.075;4.214)	1.766(0.609;3.044)	2.541(1.847;3.3)	0.568(0;2.072)
Seed mass	0.255	0	(0.186;0.327)	2.621(0.346;5.397)	1.042(0.2;1.976)	3.688(3.125;4.269)	1.753(0.685;3.026)	2.507(1.798;3.265)	0.546(0;1.933)
**Scarification**									
Main effect	-0.182	0.079	(-0.389;0.013)	2.501(0.393;5.293)	1.113(0.302;2.046)	3.874(3.293;4.434)	1.627(0.504;2.772)	2.25(1.616;2.981)	0.584(0;2.125)
Mediterranean	0.112	0.218	(-0.075;0.282)						
Habitat-specialists	-0.111	0.228	(-0.291;0.056)	2.435(0.343;5.126)	1.135(0.223;2.038)	3.886(3.299;4.45)	1.592(0.629;2.789)	2.263(1.572;2.976)	0.538(0;2.208)
Seed mass	0.038	0.297	(-0.037;0.11)	2.667(0.394;5.526)	1.021(0.186;1.924)	3.851(3.263;4.413)	1.673(0.576;2.878)	2.331(1.611;3.003)	0.555(0;2.087)
**Fire**									
Main effect	-0.163	0.128	(-0.378;0.042)	2.525(0.348;5.309)	1.025(0.217;1.946)	3.856(3.247;4.448)	1.606(0.492;2.75)	2.279(1.626;2.998)	0.571(0;2.386)
Mediterranean	-0.091	0.334	(-0.26;0.092)						
Habitat-specialists	0.091	0.317	(-0.098;0.262)	2.494(0.319;5.196)	1.046(0.238;2.004)	3.869(3.278;4.446)	1.578(0.516;2.709)	2.277(1.594;2.995)	0.571(0;2.264)
Seed mass	-0.14	0.016	(-0.249;-0.025)	2.559(0.402;5.274)	1.043(0.177;1.876)	3.851(3.216;4.377)	1.717(0.652;2.961)	2.289(1.64;3.015)	0.738(0;2.151)

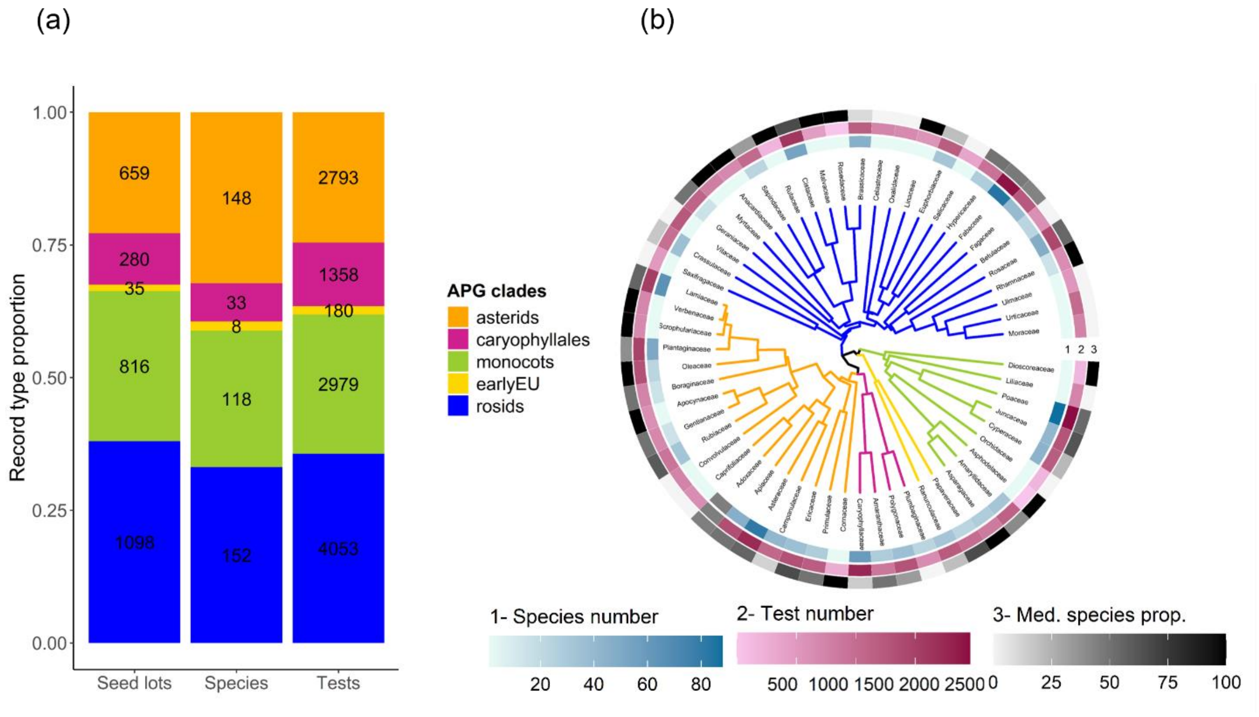
(a)
(b)
Record type proportion
1.00
0.75
0.50
0.25
0.00
659
280
35
816
1098
148
33
8
118
152
2793
1358
180
2979
4053
Seed lots
Species
Tests
APG clades
asterids
caryophyllales
monocots
earlyEU
rosids
Brassicaceae
Resedaceae
Malvaceae
Cistaceae
Rutaceae
Sapindaceae
Anacardiaceae
Myrtaceae
Geraniaceae
Vitaceae
Crassulaceae
Saxifragaceae
Celastraceae
Oxalidaceae
Linaceae
Euphorbiaceae
Salicaceae
Hypericaceae
Fabaceae
Fagaceae
Betulaceae
Rosaceae
Rhamnaceae
Ulmaceae
Urticaceae
Moraceae
Lamiaceae
Verbenaceae
Scrophulariaceae
Plantaginaceae
Oleaceae
Boraginaceae
Apocynaceae
Gentianaceae
Rubiaceae
Convolvulaceae
Caprifoliaceae
Adoxaceae
Apiaceae
Asteraceae
Campanulaceae
Ericaceae
Primulaceae
Cornaceae
Caryophyllaceae
Amaranthaceae
Polygonaceae
Plumbaginaceae
Ranunculaceae
Papaveraceae
Asparagaceae
Amaryllidaceae
Asphodelaceae
Orchidaceae
Cyperaceae
Juncaceae
Poaceae
Liliaceae
Dioscoreaceae
1 2 3
1- Species number
20 40 60 80
2- Test number
500 1000 1500 2000 2500
3- Med. species prop.
0 25 50 75 100

Figure S1.

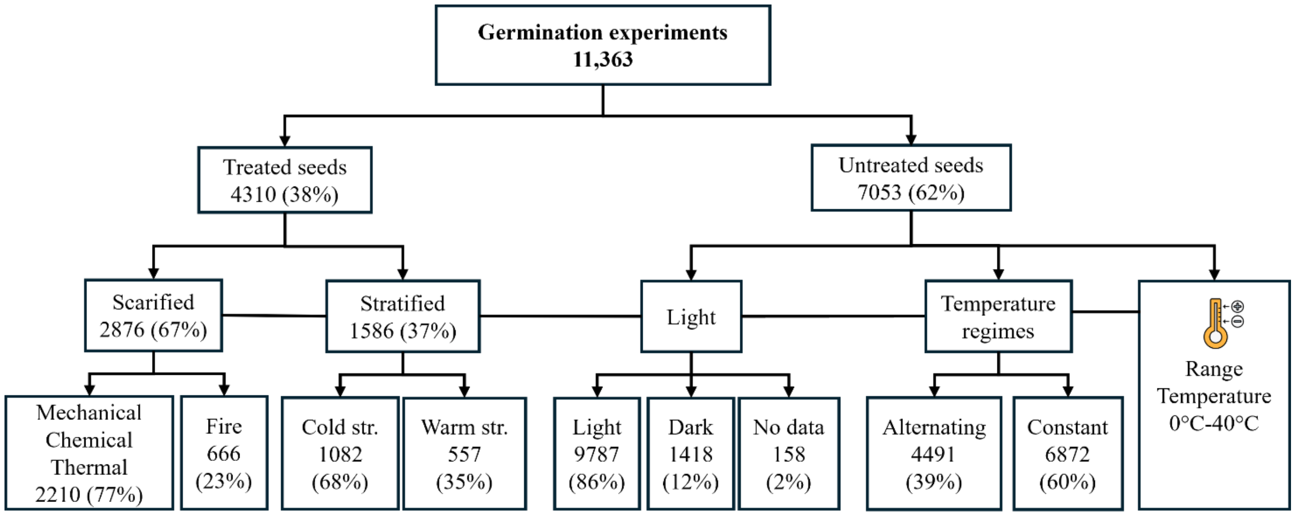
Germination experiments
11,363
Treated seeds
4310 (38%)
Untreated seeds
7053 (62%)
Scarified
2876 (67%)
Stratified
1586 (37%)
Light
Temperature regimes
Range Temperature
0°C-40°C
Mechanical
Chemical
Thermal
2210 (77%)
Fire
666
(23%)
Cold str.
1082
(68%)
Warm str.
557
(35%)
Light
9787
(86%)
Dark
1418
(12%)
No data
158
(2%)
Alternating
4491
(39%)
Constant
6872
(60%)

Figure S2

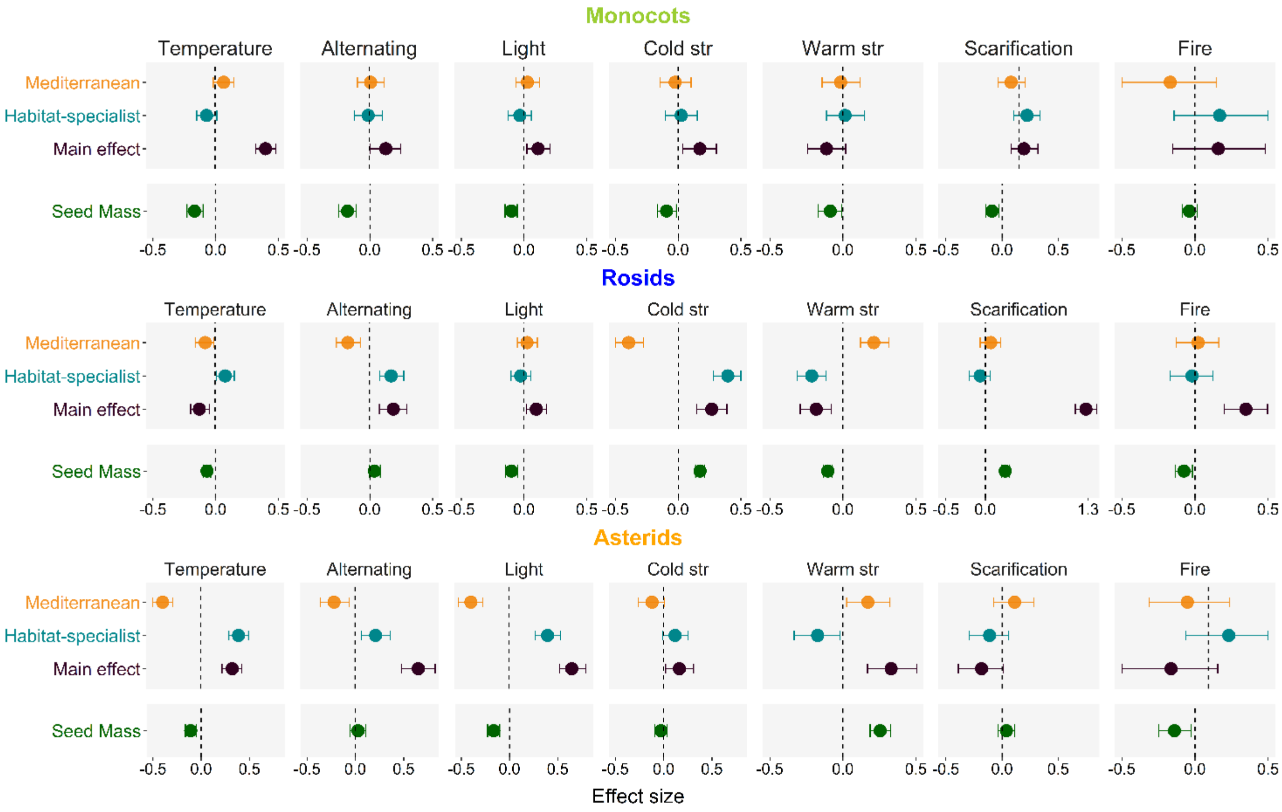
Monocots
Temperature
Alternating
Light
Cold str
Warm str
Scarification
Fire
Mediterranean
Habitat-specialist
Main effect
Seed Mass
-0.5
0.0
0.5
Rosids
1.3
Asterids
Effect size

Figure S3

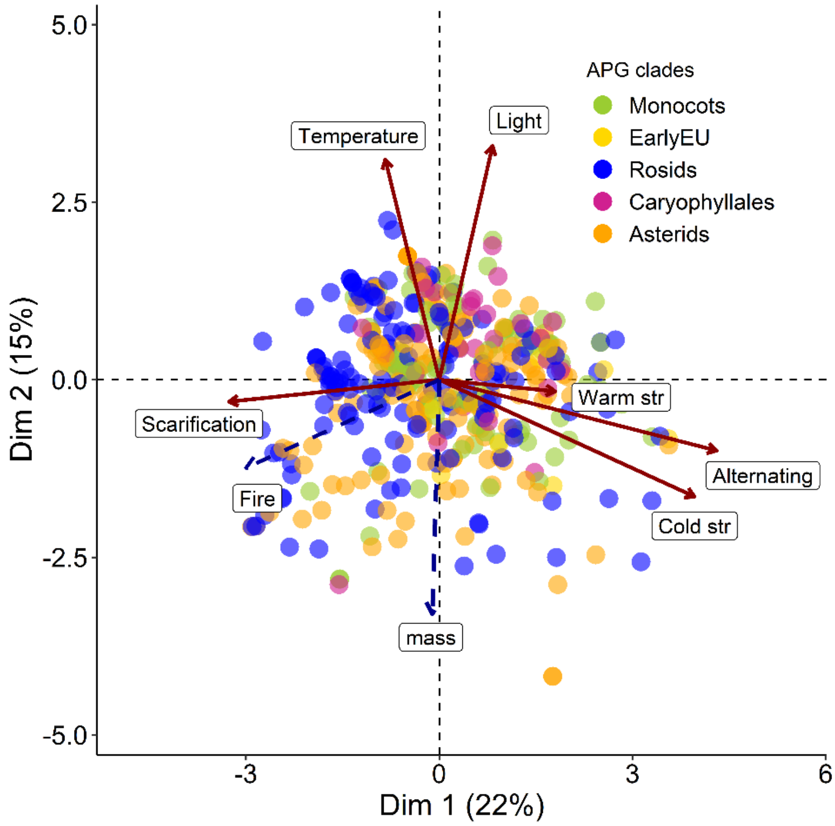

APG clades
Monocots
EarlyEU
Rosids
Caryophyllales
Asterids
Temperature
Light
Scarification
Warm str
Alternating
Cold str
Fire
mass
5.0
2.5
0.0
-2.5
-5.0
-3
0
3
6
Dim 2 (15%)
Dim 1 (22%)

Figure S4

```
# R codes used in the study to fit the MCMCglmms models are available below.

library(MCMCglmm)
# This script shows as a sample the code to fit the MCMCglmms models.

# MCMCglmm analyses
## Binomial models
priors <- list(R = list(V = 1, nu = 50),
               G = list(G1 = list(V = 1, nu = 1, alpha.mu = 0, alpha.V = 500),
                        G2 = list(V = 1, nu = 1, alpha.mu = 0, alpha.V = 500),
                        G3 = list(V = 1, nu = 1, alpha.mu = 0, alpha.V = 500),
                        G4 = list(V = 1, nu = 1, alpha.mu = 0, alpha.V = 500),
                        G5 = list(V = 1, nu = 1, alpha.mu = 0, alpha.V = 500)))

# Read the phylogenetic tree ####
phangorn::nnls.tree(cophenetic(ape::read.tree("data/seedArc_treeV0.tree")),
                    ape::read.tree("data/seedArc_treeV0.tree"), method = "ultrametric") -> nnls
nnls$node.label <- NULL
nnls.a<- drop.tip(nnls, unique(data$animal))
nnls<- drop.tip(nnls, nnls.a$tip.label)

nite = 500000
nthi = 100
nbur = 100000

# Models ####
model.par <-c(
  "scale(Tmean)+scale(Tmean):scale(p1)","scale(Tmean)+scale(Tmean):scale(p2b)",
  "scale(Alternating)+scale(Alternating):scale(p1)","scale(Alternating)+scale(Alternating):scale(p2b)",
  "scale(Scarification)+scale(Scarification):scale(p1)","scale(Scarification)+scale(Scarification):scale(p2b)",
  "scale(cold_str)+scale(cold_str):scale(p1)","scale(cold_str)+scale(cold_str):scale(p2b)",
  "scale(warm_str)+scale(warm_str):scale(p1)","scale(warm_str)+scale(warm_str):scale(p2b)",
  "scale(Fire)+scale(Fire):scale(p1)","scale(Fire)+scale(Fire):scale(p2b)")

### List of random factors ####
random_factors <- list(
  c("animal", "ID", "id_test", "doi", "seedlot", "substrate"),
  c("animal", "ID", "id_test", "doi", "seedlot", "substrate"),
  c("animal", "ID", "id_test", "doi", "seedlot", "substrate"),
  c("animal", "ID", "id_test", "doi", "seedlot", "substrate"),
  c("animal", "ID", "id_test", "doi", "seedlot", "substrate"),
  c("animal", "ID", "id_test", "doi", "seedlot", "substrate"),
  c("animal", "ID", "id_test", "doi", "seedlot", "substrate"),
  c("animal", "ID", "id_test", "doi", "seedlot", "substrate"),
  c("animal", "ID", "id_test", "doi", "seedlot", "substrate"),
  c("animal", "ID", "id_test", "doi", "seedlot", "substrate"),
  c("animal", "ID", "id_test", "doi", "seedlot", "substrate"),
  c("animal", "ID", "id_test", "doi", "seedlot", "substrate"))

# RUN MODELS global p1 and p2  ####
library(doParallel); library(foreach)

parallel::detectCores()
n.cores <- 2
my.cluster <- parallel::makeCluster(n.cores, type = "PSOCK")
doParallel::registerDoParallel(cl = my.cluster)
```

```
clusterEvalQ(my.cluster, {
  library(MCMCglmm)
})

clusterExport(my.cluster, varlist = c("data", "nnls", "nite", "nthi", "nbur", "priors", "model.par",
"random_factors"))
model.res <- foreach(i = 1:length(model.par), .packages = "foreach") %dopar% {
  fixed <- as.formula(paste("cbind(Germinated, Germinable-Germinated) ~", model.par[i], sep = ""))
  random <- random_factors[[i]]

  mm <- MCMCglmm(fixed = fixed,
               random = as.formula(paste("~", paste(random, collapse = "+"))),
               family = "multinomial2", pedigree = nnls, prior = priors, data = data,
               nitt = nite,thin = nthi,burnin = nbur,verbose = FALSE
  )
}
parallel::stopCluster(cl = my.cluster)
```
